# Challenges for HIV Pre-Exposure Prophylaxis among Men Who Have Sex with Men in the United States

**DOI:** 10.1371/journal.pmed.1001286

**Published:** 2012-08-21

**Authors:** Gordon Mansergh, Beryl A. Koblin, Patrick S. Sullivan

**Affiliations:** 1 Division of HIV/AIDS Prevention, Centers for Disease Control and Prevention, Atlanta, Georgia, United States of America; 2 Laboratory of Infectious Disease Prevention with the New York Blood Center, New York, New York, United States of America; 3 Rollins School of Public Health, Emory University, Atlanta, Georgia, United States of America

## Abstract

In light of recent research, Gordon Mansergh and colleagues discuss barriers to effective implementation of HIV pre-exposure prophylaxis for men who have sex with men.

**Summary Points**

- Pre-exposure prophylaxis (PrEP) with anti-retroviral (ARV) medications is partially efficacious for preventing HIV infection among men who have sex with men (MSM) and heterosexuals.
- As PrEP becomes available and prescribed for use among MSM a better understanding of willingness to use PrEP and avoidance of condom use are needed so that behavioral programs and counseling may be enhanced for maximum benefit.
- Targeted messaging will be needed about ARV prophylaxis for various at risk populations, but the general message should be that condoms continue to be the most effective way to prevent HIV transmission through sex and that PrEP is an additional biomedical intervention.
- As new effective biomedical intervention methods, such as PrEP, become available language about “protected” and “unprotected” sex, which used to exclusively mean condom use, will need to adapt.

Pre-exposure prophylaxis (PrEP) to prevent HIV infection with anti-retroviral (ARV) medications was found to be partially efficacious among men who have sex with men (MSM) [1] and heterosexuals [2],[3]. Other studies have provided information about potential uptake of PrEP among MSM, including factors associated with use and sharing of HIV medications before [4] and after [5] ARV efficacy was known. In a study of high-risk, substance-using MSM in four United States cities conducted prior to the release of efficacy trial results, black and Latino (versus white) MSM were more willing to use a less effective PrEP product in order to avoid condom use [6]; further, high-risk MSM with less education reported more non-prescribed, pre-efficacy ARV use (by HIV-negative men) and sharing of ARVs with sex partners (by HIV-positive men) to prevent HIV infection [4]. In an Internet study of US MSM immediately following release of the efficacy trial results among MSM, black and Latino (versus white) MSM were more willing to use PrEP after efficacy was known [5].

Colleagues [7]–[9] have identified important challenges relating to the implementation of PrEP, including specialist and generalist physician willingness to prescribe PrEP based on trial results [10]. These issues will become even more prominent due to the recent recommendation of the US Food and Drug Administration (FDA) Advisory Committee to approve emtricitabine/tenofovir disoproxil fumarate (TDF/FTC or Truvada) for use as PrEP among sexually active adult men and women. This essay addresses PrEP implementation challenges for MSM and their communities in the US.

## Challenges

PrEP implementation among MSM poses several challenges, including (a) understanding of PrEP use and use preferences; (b) PrEP implementation costs to individuals and the health care system, and the associated epidemiological impact; (c) effective messaging about PrEP to various MSM-related audiences; and (d) implications of PrEP on the dialogue and language for research and PrEP use in practice.

### Understanding PrEP Use and Preferences among MSM

Prior to known efficacy, ARV use and sharing for HIV prophylaxis among MSM was minor [11]. In a large sample of HIV-negative substance-using MSM [4] in four US cities (Chicago, Los Angeles, New York, San Diego), a group at substantial HIV risk, only 2% reported PrEP and 4% post-exposure prophylaxis (PEP) use at a time preceding known efficacy of ARV use for prophylaxis of HIV infection. Among HIV-positive MSM in the study, 2.5% and 4% reported sharing ARV drugs with partners for PrEP and PEP, respectively. In a separate analysis of the data and focusing on self-reported efficacy level needed in order to forego condom use, substantial proportions of the HIV-negative men [6] were willing to have anal sex without a condom while taking PrEP (28% for receptive and 51% for insertive), given an efficacy level at or below the range of efficacy trial findings among MSM (i.e., 44% efficacy overall for oral PrEP, up to a 73% efficacy with 90% adherence in the iPrEX trial [1]). In an Internet study of US MSM conducted in early 2011, soon after efficacy trial results among MSM were announced [5], 83% of the HIV-negative men reported that they were likely to use an oral PrEP product at 44% efficacy, the overall efficacy level found in the trial [1]. The demand for PrEP could be relatively high, depending on access, eligibility, and cost coverage. Racial/ethnic minority MSM may be particularly willing to use PrEP, and could especially benefit from its use, given extremely high HIV prevalence and incidence levels among black and Latino MSM populations [9]. As ARVs become increasingly available by prescription for HIV-negative and HIV-positive MSM, non-prescribed use of ARVs by HIV-negative men and sharing by men with HIV may increase.

More surveillance and assessment is needed to monitor and better understand, among other issues, reasons for differential willingness to use PrEP and avoidance of condom use among MSM—so that behavioral programs and counseling may be enhanced for maximum benefit for both ARV prophylaxis and condom use. Ongoing community-level behavioral and clinical research is needed to determine the prevalence and effectiveness of intermittent dosing; behavioral risk substitution for condom use, including use of seroadaptive behaviors (e.g., HIV serosorting, behavioral positioning as a lower risk insertive versus receptive partner); preferences about PrEP formulation (e.g., oral, topical); provider knowledge, perceptions, and clinical behavior; and PrEP costs and access through different service provision settings.

PrEP should be prescribed and clinically monitored by a health care professional to ensure appropriate use and effectiveness ([12]; US Centers for Disease Control and Prevention [CDC] full guidelines forthcoming), which includes medication adherence and regular HIV testing. Trial data indicate that good adherence is critical for high PrEP efficacy [1] and innovative approaches to promote PrEP adherence that are easily implemented in clinical and community settings are needed [13], including alternatives to oral administration of medication (e.g., rectal gel). Concerns about acquisition and transmission of drug-resistant HIV strains have also been raised [13], particularly if PrEP is being applied after HIV infection but before detectable infection, when a more rigorous ARV treatment should be employed. Further, monitoring is needed to capture both prescribed and non-prescribed use of ARV for HIV prophylaxis to better understand community-level utilization of PrEP and PEP, and to address the clinical, behavioral, and epidemiological implications of ARV use to prevent HIV transmission in MSM populations.

### PrEP Costs and the Related Epidemiological Impact

Issues of cost to the individual and the health care system are central to PrEP implementation [14],[15]. There continue to be waiting lists for ARV treatment in some communities due to limited funding; large segments of HIV-negative persons will not have insurance or the direct payment capability to pay for PrEP. Drug manufacturers, insurance companies, and federal agencies must work to close gaps in access, particularly among resource-poor MSM with limited access to HIV prevention and health care.

ARV coverage for HIV-negative and -positive individuals has serious implications for HIV epidemiology. Mathematical models generally focus on efficacy, coverage, and inadvertent taking of PrEP by newly HIV-infected persons before diagnosis, and have demonstrated that ARV resistance could be a concern if PrEP is not monitored for HIV infection systematically [14],[15]. Others have assessed potential cost-effectiveness of PrEP among high-risk groups such as MSM [16]; however, more cost analysis and epidemiological modeling is needed to determine implementation scenarios for optimal health benefit.

### PrEP Information and Messaging for Various MSM-Related Audiences

Targeted educational messages will be needed about ARV prophylaxis for various populations (Table 1). The general message should be that condoms continue to be the most effective way to prevent HIV transmission and acquisition through sex and that PrEP is an additional biomedical intervention that can provide protection from HIV infection. Multi-level prevention messages will need to be developed in order to maximize overall protective effects addressing key audiences of HIV-negative MSM highly affected by HIV (e.g., black and Latino MSM), HIV-negative MSM not using other prevention methods (e.g., condoms), HIV-positive MSM on ARVs (because they might consider sharing ARVs), and health care providers. For MSM and their providers, clear information and guidelines are needed about PrEP efficacy, risks of side effects and other PrEP use complications, the need for ongoing HIV testing and PrEP monitoring by providers, and the importance of adherence to prescribed PrEP [7]–[9]. Critical distinctions between PrEP and PEP and the appropriate use of each should also be addressed. At broader MSM community levels and for providers, information regarding for whom PrEP is most suitable should be provided. Specific PrEP guidelines are being developed by CDC and interim guidelines currently exist [10]; however, determination of the appropriateness of PrEP will ultimately be a mutual decision made between the patient and provider. For all of the audiences noted above, information on the dangers of sharing PrEP with others should address the risk of medication ineffectiveness and complications when PrEP is not directly prescribed and monitored by a qualified health care provider [7],[8]. Lastly, nonjudgmental support from providers is needed for HIV-negative MSM on PrEP, and provider services to promote adherence and appropriate monitoring are needed for all MSM receiving ARVs.

**Table 1:** Types of targeted PrEP messaging needed for MSM and health care providers by subgroup.

Subgroup	Type of Targeted Messaging		
	For Whom PrEP Is Appropriate	PrEP Efficacy, Risks, Adherence, Monitoring	Dangers of Sharing ARVs
Health care providers of HIV-negative MSM	X	X	X
MSM communities	X		X
HIV-negative MSM highly affected by HIV:			
• Black and Latino MSM		X	X
• Substance-using MSM		X	X
• MSM not using other approaches (e.g., condoms)		X	X
ARV-taking friends and sex partners of HIV-negative MSM (e.g., HIV-positive MSM)		X	X

ARV, antiretroviral medication; MSM, men who have sex with men; PrEP, pre-exposure HIV prophylaxis.

### Dialogue and Language Issues Associated with PrEP

As we transition from an era of one effective biomedical intervention method (i.e., condoms) to an era of multiple efficacious interventions (i.e., condoms, ARV prophylaxis, and ARV treatment as prevention [17]), language about “protected” and “unprotected” sex will need to adapt. Protection from HIV infection previously meant condom use; however, protection in the future could also include oral PrEP, PEP, topical agents, or other products. This is also an opportune time to have a more explicit discussion about the efficacy and effectiveness of both methods. The research literature suggests that condoms are generally in the range of 80%–87% effective in reducing HIV transmission in studies of vaginal use among heterosexuals [14],[15]. Given efficacy trial results among MSM, PrEP can be 44% efficacious among MSM and 73% efficacious with high (90%) adherence. However, other host and partner factors are critically important in the equation (e.g., viral load, immune system functioning, number and intensity of exposures, severity of PrEP side effects). Taken together, condom use continues to be strongly recommended for the prevention of sexual transmission of HIV for all MSM, and PrEP may provide additional protection for some very high-risk MSM, determined jointly by MSM and their health care providers. However, when researchers and practitioners discuss protective action and its effect on HIV transmission, we must be clear in assessing condom use, and PrEP uptake and adherence. For example, MSM studies will need to assess separately for sexual event-level condom use and adherence and PrEP use and adherence, and include or control for each other in analysis.

Language becomes complicated when capturing PrEP and PEP—and the potential use of both by some men—in practice. Taking ARVs for prophylaxis before sexual exposure is considered PrEP. However, if a high-risk HIV-negative person who has recently engaged in sexual risk behavior is prescribed and is adherent to PrEP, but still becomes infected with HIV, PrEP becomes a suboptimal form of treatment. Over time, success of ARV prophylaxis may be due to administration before and/or after sexual exposures. The choice of approach—PrEP as a continuous approach, or PEP as an episodic approach—is contingent upon close monitoring and open discussions between providers and patients. As PrEP and PEP use become more common in the future, distinguishing between PrEP and PEP could become complex for individuals, and thus for research based on behavioral self-report. Detailed assessments will be needed to measure the complexity of ARV use over time.

## Next Steps

Although PrEP will likely be inaccessible to many US MSM in the near future because of prohibitive cost to individuals and the health care system, some HIV-negative MSM may be prescribed ARVs and other MSM may inappropriately obtain ARVs from friends or sex partners to prevent HIV infection. Over time, access to PrEP could become a reality for many MSM, particularly in high-income countries, as availability increases and costs decrease. Given the emergence of this prophylaxis option, public health officials and providers are challenged to address community-level monitoring of prescribed and non-prescribed PrEP use in addition to condom use; develop effective multiple messages about condom use and PrEP for MSM and for their health care providers; disseminate information about the hazards of sharing ARV medications; and develop language about HIV prevention and risk reduction that has historically focused almost exclusively on condom use for sexually active MSM. As other efficacious HIV prevention interventions become available (e.g., topical antiviral products) [17], lessons learned from PrEP implementation can be applied to roll out of those approaches as well.

## Abbreviations

ARV: anti-retroviral
MSM: men who have sex with men
CDC: Centers for Disease Control and Prevention
PEP: HIV post-exposure prophylaxis
PrEP: HIV pre-exposure prophylaxis
